# Supplementary material for: Patients with Systemic Juvenile Idiopathic Arthritis (SJIA) Show Differences in Autoantibody Signatures Based on Disease Activity
Source: Biomolecules. 2023 Sep 15;13(9):1392. doi: 10.3390/biom13091392 (PMC10527260; doi:10.3390/biom13091392)
Supplement: Supplementary file 1 [file biomolecules-13-01392-s001.zip › supplementary_figures_KRAINER.pdf]

Supplementary Figures

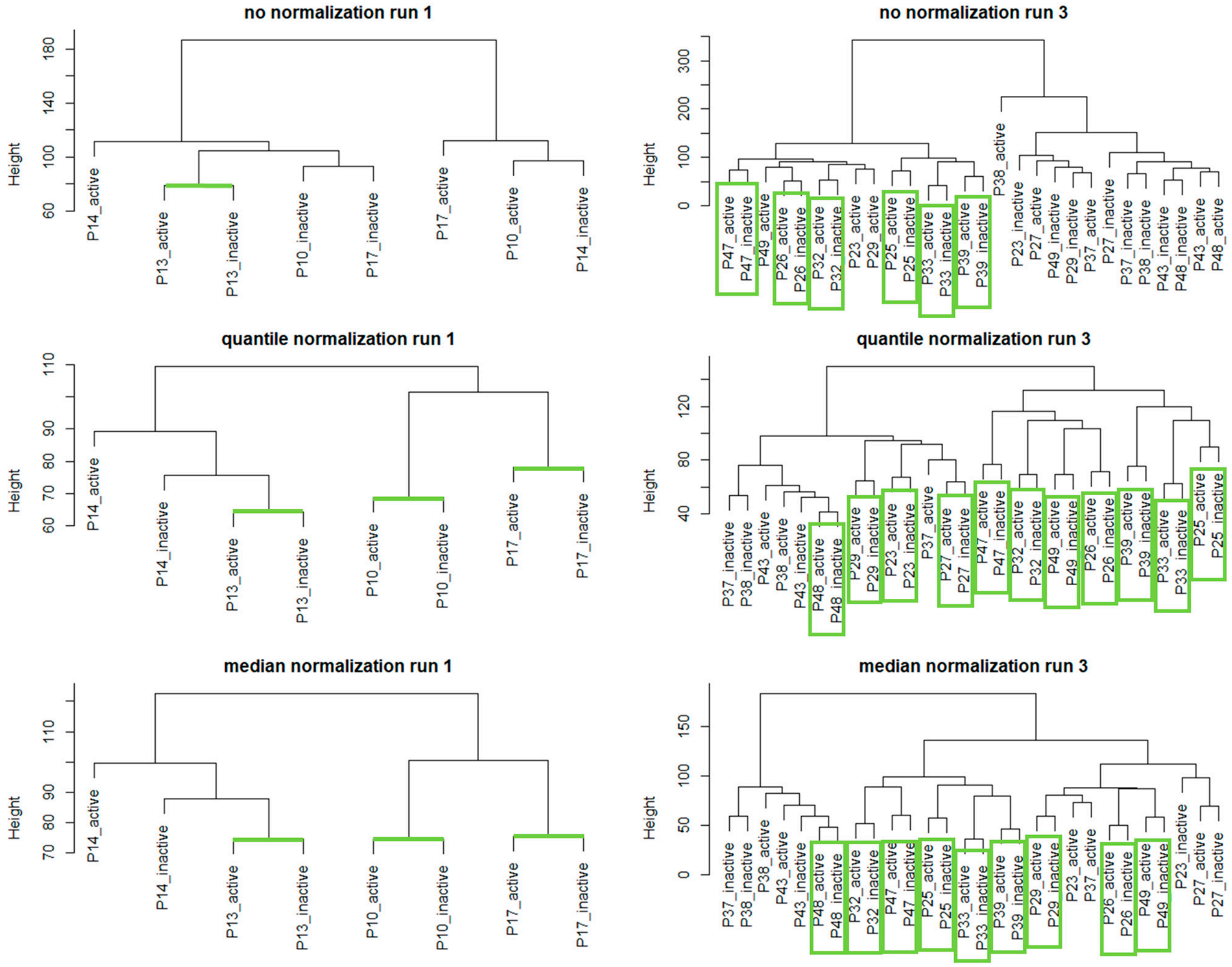

Supp. Fig. S1: Hierarchical clustering of the 36 paired samples during analysis run 1 (left panel) and run 3 (right panel). In total 18 paired samples were analyzed,without normalization 7 out of 14 pairs cluster with each other (top row), quantile normalization resulted in 14 pairs clustering (middle row), and median normalization in 12 pairs clustering (bottom row)

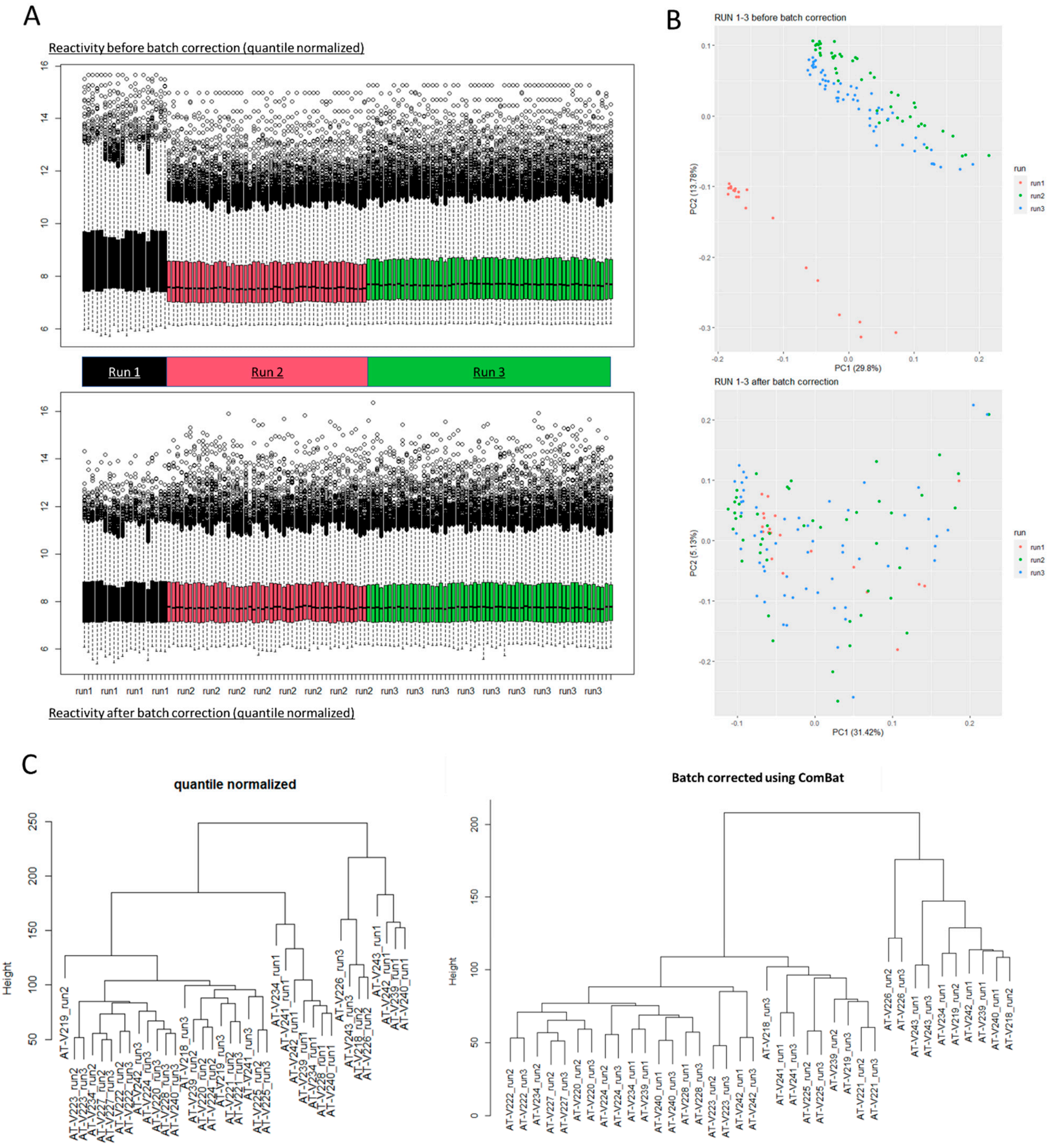

Supp. Fig. S2: A – shows the microarray intensity of the analysis runs performed on three days, where run 1 has a lower intensity compared to run 2 and run 3. B – PCA of the samples before batch correction (top) and after batch correction (bottom) showing the removal of the effect. C – hierarchical clustering of the healthy controls processed twice or three times on multiple analysis days. Without batch correction, 5 out of 21 healthy controls analyzed on multiple analysis runs clustered together during hierarchical clustering. After batch correction 11 healthy controls cluster together, for two healthy controls, analyzed on all three analysis runs, two of three clustered together showing the successful effect of the batch correction step.

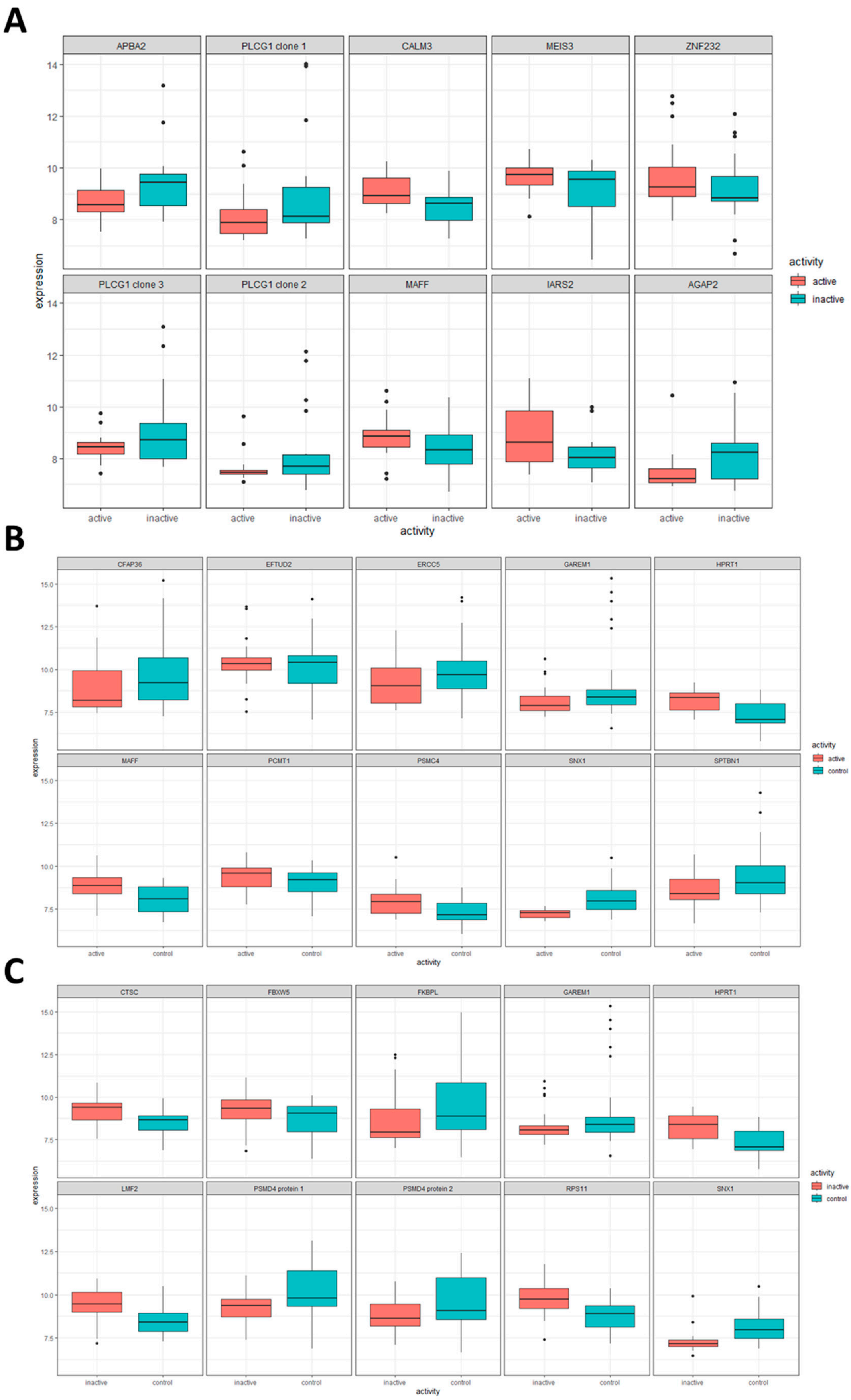

Supp. Fig. S3: Top 5 higher/lower reactive DIRAGs between the comparison of A – active SJIA vs. inactive SJIA paired; B – active SJIA vs. healthy controls; C – SJIA inactive vs. healthy controls.

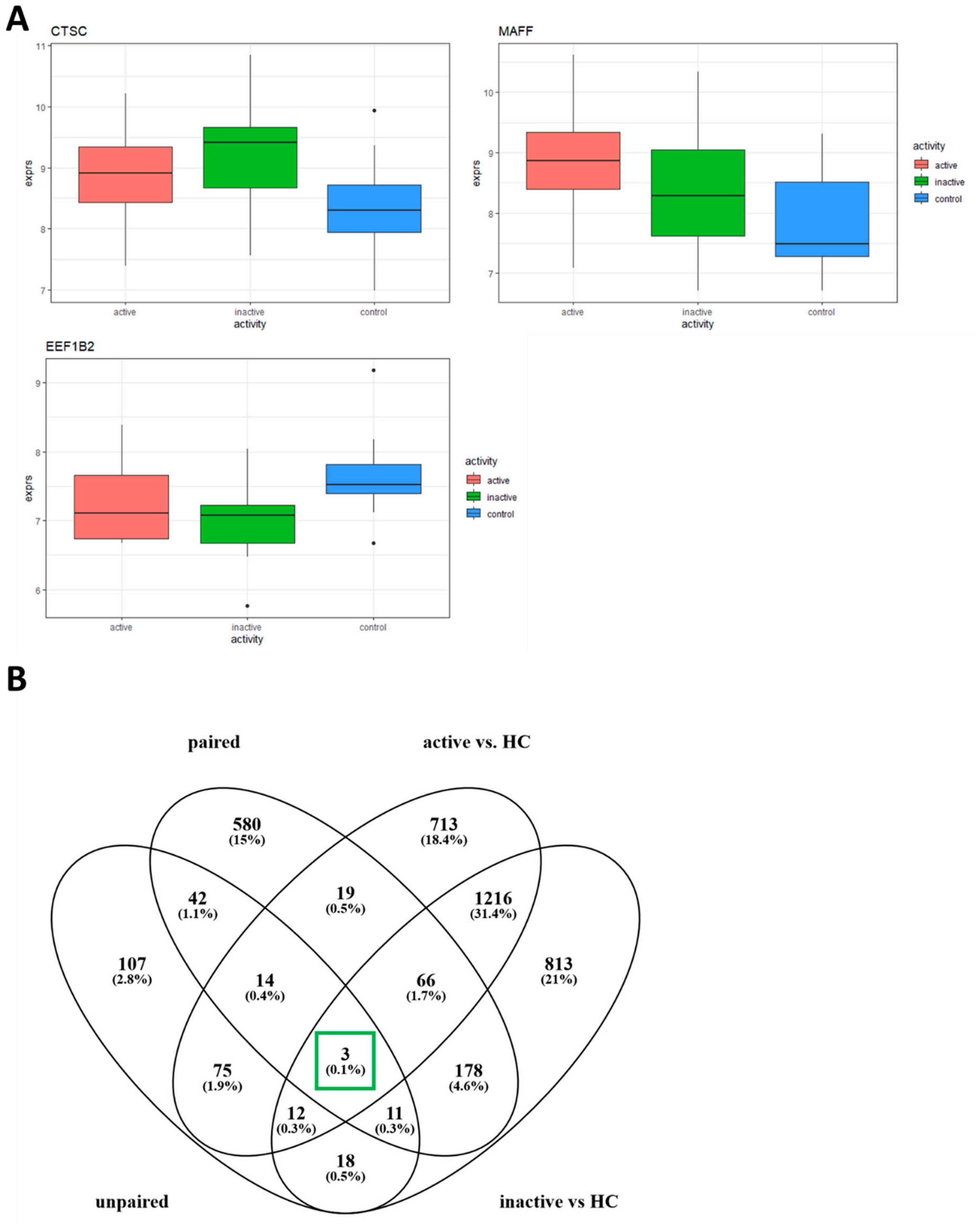

Supp. Fig. S4: A – Venn diagram of DIRAGs in all three group comparison, showing 3 overlaps. B - D Differentially reactive autoantigens overlapping in all three group comparisons.
